# Impact of underlying medical conditions and medications on edema development in alopecia patients treated with low-dose oral minoxidil: A retrospective study

**DOI:** 10.1016/j.jdin.2025.05.010

**Published:** 2025-06-16

**Authors:** Deesha Desai, Ambika Nohria, Anna Brinks, Carli Needle, Michelle Sikora, Soutrik Mandal, Jerry Shapiro, Avrom S. Caplan, Michael Garshick, Kristen I. Lo Sicco

**Affiliations:** a University of Pittsburgh School of Medicine, Pittsburgh, Pennsylvania; b The Ronald O. Perelman Department of Dermatology, NYU Grossman School of Medicine, New York, New York; c New York Medical College, Valhalla, New York; d Department of Population Health, NYU Grossman School of Medicine, New York, New York; e Leon H. Charney Division of Cardiology, New York University Grossman School of Medicine, New York, New York

**Keywords:** alopecia, edema, hair loss, minoxidil, safety

*To the Editor:* Low-dose oral minoxidil (LDOM) has transformed alopecia treatment. However, its vasodilatory effects can lead to complications including the development of edema, and rarely, pericardial and pleural effusions necessitating hospitalization.^1^ A gap remains in understanding the influence of underlying factors on edema development and severity. Our study investigates the impact of medical conditions and medications predisposing patients to edema on the risk of edema development in alopecia patients taking LDOM.

A retrospective analysis (January 1, 2009-August 28, 2023) included alopecia patients prescribed LDOM with at least 2 dermatology visits at New York University Langone Health. Statistical significance was determined using Fisher’s exact test.

The cohort comprised 174 patients (36.8% males and 63.2% females) with an average age of 46.6 years (range: 18-84 years). Of these, 87.4% had nonscarring alopecia (66.1% androgenetic alopecia, 19.5% alopecia areata, and 13.2% telogen effluvium) and 20.7% had scarring alopecia (11.5% lichen planopilaris, 7.5% frontal fibrosing alopecia, 1.7% chronic traction alopecia, and 1.1% central centrifugal cicatricial alopecia).

Medications that may also cause edema are oral birth control (10.9%) and nonsteroidal anti-inflammatory drugs (8.0%). Medical conditions that may cause edema are thyroid disease (13.2%) and obstructive sleep apnea (4.6%). Importantly, patients did not have significant cardiovascular or renal conditions that increase the risk of LDOM side effects.

Edema occurred in 11 patients (6.3%); it was observed independently in the ankles (36.4%), midsection (18.2%), and periorbital region (18.2%) (Table I). No cases required hospitalization, and there were no reports of pericardial or pleural effusions. The average time to edema was 151.6 days (range: 46-308). The median LDOM dose that resulted in edema was 1.3 mg (range: 0.625-5 mg).

**Table I:** Clinical characteristics and outcomes of patients with edema on low-dose oral minoxidil

Cohort	Diagnosis	Sex	LDOM dose in mg	Edema-predisposing medications	Edema-predisposing medical conditions	Location of edema	Outcome
1	AA	Female	0.625 mg	None	None	Not listed	Edema resolution, no required medication adjustment
2	AGA	Female	1.25 mg	NSAIDs	Lymphedema	Ankle	Edema remained, no required medication adjustment
3	AGA	Female	0.625 mg	None	Thyroid disease	Ankle	Edema resolution with addition of spironolactone 50 mg
4	AGA/TE	Female	0.625 mg	Nifedipine	Chronic lung disease	Ankle	Edema remained, no required medication adjustment
5	TE	Female	2.5 mg	None	Lymphedema	Not listed	Edema remained, no required medication adjustment
6	AGA/TE	Female	1.25 mg	None	None	Midsection/waistline	Edema remained, no required medication adjustment
7	AA	Male	2.5 mg	Oral steroids	None	Facial	Edema remained, no required medication adjustment
8	AGA/TE	Male	5 mg	None	None	Periorbital	Edema resolution, no required medication adjustment
9	AGA/TE	Male	2.5 mg	None	None	Midsection/waistline	Edema resolution, no required medication adjustment
10	AGA	Female	1.25 mg	Birth control pills	Liver failure, chronic lung disease, obstructive sleep apnea	Ankle	Required dose reduction to 0.625 mg and addition of spironolactone 50 mg, edema resolution
11	AA	Female	1.25 mg	Birth control pills	None	Periorbital	Required medication discontinuation, edema resolution thereafter

*AA*, Alopecia areata; *AGA*, androgenetic alopecia; *LDOM*, low-dose oral minoxidil; *NSAIDs*, nonsteroidal anti-inflammatory drugs; *TE*, telogen effluvium.

No statistically significant associations were noted between edema occurrence and the use of an edema-predisposing medication or medical condition. Similarly, no significant associations were observed between edema development and sex or LDOM dose (Table II).

**Table II:** Associations between patient characteristics, predisposing factors, oral minoxidil dose, and edema development

	No edema (*N* = 163)	Edema (*N* = 11)	*P* value
Association between predisposing edema medication and edema side effect			
No predisposing edema medication	62.6% (102)	54.5% (6)	.750
Predisposing edema medication	37.4% (61)	45.5% (5)	
Association between predisposing edema medical condition and edema side effect			
No predisposing edema medical condition	74.8% (122)	54.5% (6)	.162
Predisposing edema medication condition	25.2% (41)	45.5% (5)	
Association between sex and edema side effect			
Male	37.4% (61)	27.3% (3)	.748
Female	62.6% (102)	72.7% (8)	
Association between LDOM dosage and edema side effect			
LDOM ≥2.5	37.4% (61)	36.4% (4)	.748
LDOM <2.5	62.6% (102)	63.6% (7)	

*LDOM*, Low-dose oral minoxidil.

Among edema cases, 81.2% experienced no LDOM dosage change or discontinuation (55.6% reported edema tolerability; 44.4% achieved resolution), 9.1% required dose adjustment with eventual resolution, and 9.1% discontinued LDOM due to edema intolerability without attempting dose adjustment.

The absence of notable associations may lower provider concerns that such medications increase edema risk on LDOM. Furthermore, it may suggest that these medical conditions do not significantly impact LDOM’s pharmacological profile, supporting previously published findings showing good tolerability in systemic lupus erythematosus patients using <5 mg daily.^2^ While these findings may reassure clinicians considering LDOM for alopecia patients with such conditions, larger studies should be performed to further determine if other known contributors to edema, including body mass index, hot weather, and medication formulation, contribute to edema development.^3^

Oral minoxidil carries a box warning for the risk of developing pericardial effusion.^4^ However, our patients primarily experienced edema in the ankles, midsection, and periorbital region with no instances of pericardial or pleural effusions, or hospitalizations. This differs from previously reported rates of edema, potentially secondary to sample size.^5^ While providers may feel more assured in prescribing LDOM, it is important to consider that 5 mg is the lowest cardiovascular dose, with the potential for more severe fluid retention in at-risk populations.^2^

Study limitations include small sample size, low median oral minoxidil dose, retrospective design, patient self-reporting of edema, and reliance on chart documentation.

## Conflicts of interest

None disclosed.
